# Urethroplasty with tunica vaginalis flap for correction of a rare complication of indwelling catheterization: the kippered urethra

**DOI:** 10.1590/S1677-5538.IBJU.2020.0505

**Published:** 2020-09-02

**Authors:** Fernando S. da Silva, José Anacleto D. de Resende, Daniel S. Juraski, Luciano A. Favorito

**Affiliations:** 1 Serviço de Urologia Hospital Federal da Lagoa Rio de JaneiroRJ Brasil Serviço de Urologia, Hospital Federal da Lagoa, Rio de Janeiro, RJ, Brasil

## Abstract

**Purpose:**

Kippered urethra is a rare complication of chronic indwelling catheterization (CIC) (1). In this condition, the urethral reconstruction is a challenge owing to local tissue scarring and a paucity of adjacent healthy tissue. The use of flaps is very important to protect the suture line and avoid fistulas (2). The aim of this video is to present for the first time a technique of urethroplasty with the use of a tunica vaginalis flap (TVF) to correct a kippered urethra in a patient with neurogenic bladder and CIC.

**Materials and Methods:**

A 46 years old male, with neurogenic bladder after a spinal cord trauma in 2013 and CIC developed a kippered urethra, with urethral and skin ventral erosion in the middle portion of the penile shaft. For reconstruction, a fusiform incision was made around the urethral erosion. After dissection and mobilization of the urethral margins, urethral tubularization was performed in a 2 planes continuous suture of its margins with 4-0 PDS. Luminal diameter was calibrated with a 16 fr Foley catheter. The next step was the access of the left testicle by a subcutaneous tunnel and confection of a 5cm vascularized TVF. This tissue was used to cover the urethral suture.

**Results:**

The patient was discharged after 48h and had no complications. Foley catheter was removed in the 10th postoperative day. Uroflowmetry was not performed because the patient didn’t have spontaneous miction. After 8 months of follow-up the patient didn’t report difficulties in intermittent self catheterisation. There were no changes in the final aesthetic of the penis, except for the scar. Regarding the patient’s sexual activity, as he maintained sexual abstinence due to erectile dysfunction and paraplegia, this outcome was not evaluated.

**Discussion and Conclusion:**

Penile urethral erosions are rare, but a potential complication of CIC (3). A standardised approach for repairing is not yet available. There is a high concern about the superposition of the suture lines, which could increase the risk of fistula formation. TVF is useful for hypospadia correction (4-6), and we believe that the same results will be obtained with the use of this flap in urethral erosions. Regarding the lack of skin on the ventral side, this was not a problem in this case, considering that the primary closure was carried out without complications. We have records of 4 similar cases in which we performed the same technique. In all of them, the primary closing was possible with the foreskin itself. As far as we know, there are no reports about the use of this technique in cases of urethral erosion after CIC. In conclusion, a urethroplasty with TVF technique may be a viable method for repairing penile urethral erosions, but further studies are required.

